# Machine Learning Enabled Performance Prediction Model for Massive-MIMO HetNet System

**DOI:** 10.3390/s21030800

**Published:** 2021-01-26

**Authors:** Shuvabrata Bandopadhaya, Soumya Ranjan Samal, Vladimir Poulkov

**Affiliations:** 1 School of Engineering & Technology, BML Munjal University, Gurugram 122414, India; shuvabrata.bandopadhaya@bmu.edu.in; 2 Faculty of Telecommunications, Technical University of Sofia, 1756 Sofia, Bulgaria; sranjansamal@tu-sofia.bg

**Keywords:** 5G, B5G wireless networks, massive MIMO, HetNet, machine learning, coverage probability, area spectral density

## Abstract

To support upcoming novel applications, fifth generation (5G) and beyond 5G (B5G) wireless networks are being propelled to deploy an ultra-dense network with an ultra-high spectral efficiency using the combination of heterogeneous network (HetNet) solutions and massive Multiple Input Multiple Output (MIMO). As the deployment of massive MIMO HetNet systems involves a high capital expenditure, network service providers need a precise performance analysis before investment. The performance of such networks is limited because of presence of inter-cell and inter-tier interferences. The conventional analytic approach to model the performance of such networks is not trivial, as the performance is a stochastic function of many network parameters. This paper proposes a machine learning (ML) approach to predict the network performance of a massive MIMO HetNet system considering a multi-cell scenario. This paper considers a two-tier network in which the base stations of each tier are equipped with massive MIMO systems working in a sub 6-GHz band. The coverage probability (CP) and area spectral efficiency (ASE) are considered to be the network performance metrics that quantify the reliability and achievable rate in the network, respectively. Here, an ML model is inferred to predict the numerical values of the performance metrics for an arbitrary network configuration. In the process of practical deployments of future networks, the use of this model could be very valuable.

## 1. Introduction

To support the upcoming novel applications, such as IoT, self-driving cars, Industry 4.0, smart healthcare systems, AR/VR services, fifth generation (5G) and beyond fifth generation (B5G) wireless networks aim to achieve ultra-low latency and ultra-reliability with a multi-gigabit transmission rate [1]. The combination of heterogeneous network (HetNet) solutions and massive MIMO promises significant improvement in the physical layer performance by deploying an ultra-dense network with an ultra-high spectral efficiency [2,3]. Massive MIMO is the next generation MIMO system that significantly enhances the spectral efficiency of the communication link compared with its conventional counterpart. With this technology, a few hundred antennas are deployed at the base-station (BS) to serve a few tens of active users in same time–frequency grid [4,5]. Massive MIMO is considered a key technology for upcoming wireless generations to support 100× data rates per user and per cell by implementing adaptive beamforming and spatial multiplexing technologies with large antenna arrays [6]. HetNet is a network densification technique in which several classes of low-powered transmitters are deployed with the existing macro cells sharing the same spectrum. The low-powered small-/pico-cells are deployed to target highly concentrated user groups. The network densification process significantly improves the coverage penetration and area spectral efficiency of the network with non-uniformly distributed users [7]. By optimizing resource utilization and network performance, HetNets are going to be a principal candidate for the implementation of 5G and B5G networks [8].

For achieving customer satisfaction, which is the aim of network service providers, the latter continuously upgrade the network for maximizing the coverage probability and achievable rate for the end users. The deployment of massive MIMO HetNet systems for the upcoming network generation needs precise planning and high capital expenditure. Hence, before deployment, network service providers must optimize network parameters in order to meet the desired goal, which requires precise performance analysis. The analysis and estimation of massive MIMO system performance before practical deployment has been a major concern of research for the past few years. An asymptotic analysis of the coverage probability and sum-rate of a single-cell massive MIMO system has been presented in the literature [9]. The performance of single-cell downlink massive MIMO in terms of spectral efficiencies and link reliability using various precoding techniques has been analyzed and compared by the authors of [10]. Gao et al. have proposed a performance evaluation technique for massive a MIMO system based on propagation data [11]. Feng et al. have modeled the user-interference power distribution in a single-cell multi-user massive MIMO system using Gamma function, and formulated asymptotic deterministic equivalences for sum-rate and outage probability in terms of tight-form approximation of the model [12].

The performance of a massive MIMO system in a multi-cell scenario is limited because of the presence of inter-cell interference. A typical user in a multi-cell scenario associated with a given BS receives signals from other BSs as interference. Liang et al. have done a statistical analysis of the interference present in a massive MIMO system, considering inter-cell interference as the dominant component [13]. Li et al. have derived a large-scale approximation of the downlink signal power to interference plus noise power ratio (SINR) in a multi-cell massive MIMO system with their proposed MMSE precoder [14]. Adhikary et al. have done a tractable analysis of the interference in uplink for a large-scale antenna system [15]. The closed form outage probability of a typical user has been derived in terms of BS density and the maximum number of users served by a BS for a multi-cell massive MIMO system, assuming the BSs are distributed randomly following a Poisson point process (PPP) [16].

Similarly, in HetNet, the network performance is limited by inter-tier interference. The interference experienced by a typical user in a k-tier HetNet has been accurately modeled by the authors of [17], where each tier differs by transmitting power and cell density. Closed-form approximation has been made for the coverage probability, traffic off-loading, and sum-rate considering inter-tier interference. The analysis of HetNet with a multiple antenna system is relatively complex because of the random matrix channel. The expressions for success probability and area spectral efficiency have been formulated for MIMO heterogeneous cellular networks using a Toeplitz matrix representation [18]. With proper analysis of the interference and its cancellation, the association of massive MIMO with HetNet is a promising physical layer solution for next generation wireless networks with several-fold increases in area spectral efficiency (ASE) and coverage probability (CP) [19]. The detailed system model and performance analysis of massive MIMO with HetNet is discussed in the literature [20] and the references therein. The impact of different network settings on the virtual coverage areas for massive MIMO-enabled HetNet has been explicitly studied in the literature [20]. With a theoretic framework for massive MIMO HetNets, tractable expressions have been obtained [21] for evaluating the average achievable rate and ASE. The performance of massive MIMO HetNet systems with limited channel information is discussed in the literature [22]. A wireless backhaul-based downlink rate enhancement technique for multi-antenna HetNet is presented in the literature [23]. A tractable approach is developed for evaluation of the spectral and energy efficiency in a massive MIMO-enabled three-tier network considering the presence of eavesdroppers [24].

The performance of massive MIMO-enabled HetNet in a multi-cell scenario is limited because of the presence of both inter-cell and inter-tier interferences. Therefore, the conventional analytic approach to model the statistical behavior of overall interference is not trivial, as it needs to include many stochastic network parameters. To overcome this challenge, this paper has proposed an ML-based approach to model the massive MIMO enabled HetNet system for predicting the network performance. To the best knowledge of the authors, currently, in the literature, such an approach to predict the performance of such networks has not yet been considered.

The main focus of the work is to predict the performance of a two-tier network, in which the base stations of each tier are equipped with massive MIMO systems working in a sub 6-GHz band. This paper assumes perfect channel state information (CSI) at the transmitter. The analysis for imperfect/outdated CSI is beyond the scope of the work and may be considered in future. In this work, the coverage probability (CP) and area spectral efficiency (ASE) are considered to be the network performance metrics that quantify the reliability and achievable rate of the network, respectively. Both metrics are stochastically related with the network parameters. Two separate supervised ML models are inferred to predict the numerical values of CP and ASE from a given set of network parameters of an arbitrary network configuration. The rest of the paper is organized as follows: the system model for a multi-cell massive MIMO HetNet system is given in Section 2, the ML enabled performance prediction model in Section 3, and Section 4 concludes the paper.

## 2. System Model

### 2.1. Network Topology

**Figure 1:**
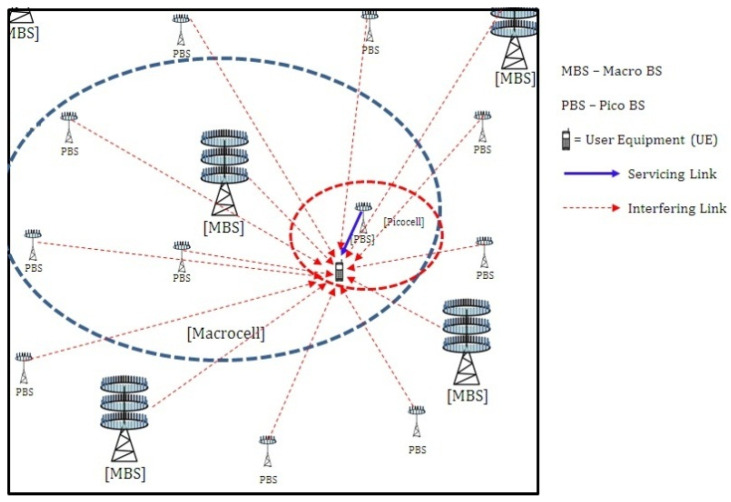
Schematic representation of a typical massive MIMO-enabled two-tier heterogeneous network (HetNet).

A HetNet system deployed in a bounded area, $A\subset {\mathbb{R}}^{2}$, with randomly placed BSs forming K tiers is considered. Each tier is distinguished by a unique transmit power, density (the average number of BSs per unit area), and antenna configuration providing service to same coverage area in the same frequency band. The spatial locations of the base stations of each tier are represented with a stochastic two-dimensional point process. For the tractable analysis, the spatial locations of the BSs of each tier ae modeled with an independent Poisson point process (PPP). $\Phi_{k}\left(A\right)∼\mathrm{PPP}\left(\lambda_{k}\right),$ *k* = 1, 2, K, where $\lambda_{k}$ is the density of the *k*-th tier, which captures the worst-case scenario [25]. In the given bounded area, $L_{k}$ numbers of *k*-th tier BSs are being deployed with a uniform transmitting power, $P_{k}$. This work considers K = 2, representing macro-base stations (MBS) acting as an umbrella cell, under which many low-powered pico-base stations (PBS) are being deployed in the vicinity of user hotspots in order to provide uniform service. The BSs of each tier are equipped with massive-MIMO transmission systems capable of serving multiple users in same time–frequency grid, considering intra-cell SDMA. Each BS of the *k*-th tier is equipped with $N_{k}$ transmit antennas, serving $R_{k}$ single antenna active users in the same time–frequency grid $\left(R_{k}<N_{k}\right)$. The schematic representation of the given model is presented in Figure 1.

### 2.2. Channel Model

In this work, both tiers are working in a sub 6-GHz band. An Orthogonal Frequency Division Multiplex (OFDM) communication system is assumed, resulting in a flat-fading channel for each sub-carrier; the channel between the *i*-th antenna in the BS of the *m*-th cell of *k*-th tier, and the *j*-th user associated with the *n*-th cell of the *l*-th tier, has been modeled with a single-tap channel coefficient [15].

(1) $$g_{\left(mi\right)k}^{\left(nj\right)l}=\sqrt{\gamma_{\left(m\right)k}^{\left(nj\right)l}}h_{\left(mi\right)k}^{\left(nj\right)l}.$$

The subscript (mi)k and superscript (nj)l represent the index of the transmitting antenna at the BS and user, respectively. $\gamma_{\left(m\right)k}^{\left(nj\right)l}$ and $h_{\left(mi\right)k}^{\left(nj\right)l}$ are the coefficients capturing the large-scale and small-scale fading effect of the channel, respectively. $\gamma_{\left(m\right)k}^{\left(nj\right)l}\in {\mathbb{R}}^{+}$ is the combination of the path attenuation, depending on the distance between user and the BS, and the shadowing effect due to the propagation environment. The value is modeled with $10log_{10}(\gamma_{m}^{n})=(-127.8-35\log d_{m}^{n}+X_{\sigma^{2}})\mathrm{dB}$, where $d_{m}^{n}$ is the Euclidean distance between the corresponding *n*-th user and the *m*-th BS, and $X_{\sigma^{2}}$ is a log-normally distributed random variable with zero mean and $\sigma^{2}$ dB variance [26]. $h_{\left(mi\right)k}^{\left(nj\right)l}\in \mathbb{C}$ is a random variable drawn from an independent Rayleigh distribution, $h_{\left(mi\right)k}^{\left(nj\right)l}∼∁ℵ\left(0,1\right).$ Considering the block-fading channel, the expected value of the channel coefficient within the coherence time is given by $E[g_{\left(mi\right)k}^{\left(nj\right)l}]=\sqrt{\gamma_{\left(m\right)k}^{\left(nj\right)l}}$. The channel coefficient vector between the BS of the *m*-th cell in the *k*-th tier, and the *j*-th user of the *n*-th cell in *l*-th tier is as follows

(2) $$g_{\left(m\right)k}^{\left(nj\right)l}=\sqrt{\gamma_{\left(m\right)k}^{\left(nj\right)l}}h_{\left(m\right)k}^{\left(nj\right)l}\in {\mathbb{C}}^{N_{k}X1}=\sqrt{\gamma_{\left(m\right)k}^{\left(nj\right)l}}{\left(h_{\left(m1\right)k}^{\left(nj\right)l},h_{\left(m2\right)k}^{\left(nj\right)l},\ldots ,h_{\left(mN_{k}\right)k}^{\left(nj\right)l}\right)}^{T}.$$

### 2.3. Interference Analysis

Using a matched filter beamformer, the transmit vector of the BS of *m*-th cell in *k*-th tier is as follows

(3) $$x_{\left(m\right)k}=\sum_{j=1}^{R_{k}}\frac{{\left[g_{\left(m\right)k}^{\left(mj\right)k}\right]}^{H}}{\left\|g_{\left(m\right)k}^{\left(mj\right)k}\right\|}s^{\left(mj\right)k}.$$

Here, $s^{\left(mj\right)k}$ is the information symbol intended for the *j*-th user in same cell. The signal received at the *j*-th associated user of the *n*-th cell in the *l*-th tier is as follows

(4) $$y^{\left(nj\right)l}=\sum_{k=1}^{K}\sum_{m=1}^{L_{k}}P_{k}g_{\left(m\right)k}^{\left(nj\right)k}x_{\left(m\right)k}+z$$

Here, z is the additive Gaussian noise, *z*$∼∁ℵ\left(0,\sigma_{z}^{2}\right)$.

(5) $$y^{\left(nj\right)l}=\sum_{m=1}^{L_{l}}P_{l}g_{\left(m\right)l}^{\left(nj\right)l}x_{\left(m\right)l}+\sum_{k=1,k\neq l}^{K}\sum_{m=1}^{L_{k}}P_{k}g_{\left(m\right)k}^{\left(nj\right)k}x_{\left(m\right)k}+z.$$

The expression of inter-tier interference is as follows

(6) $$I_{inter-tier}=\sum_{k=1,k\neq l}^{K}\sum_{m=1}^{L_{k}}P_{k}g_{\left(m\right)k}^{\left(nj\right)k}x_{\left(m\right)k}.$$

Putting Equation (6) in Equation (5),

(7) $$y^{\left(nj\right)l}=P_{l}g_{\left(n\right)l}^{\left(nj\right)l}x_{\left(n\right)l}+\sum_{m=1,m\neq n}^{L_{l}}P_{l}g_{\left(m\right)l}^{\left(nj\right)l}x_{\left(m\right)l}+I_{inter-tier}+z.$$

The expression of inter-cell interference is as follows

(8) $$I_{inter-cell}=\sum_{m=1,m\neq n}^{L_{l}}P_{l}g_{\left(m\right)l}^{\left(nj\right)l}x_{\left(m\right)l}.$$

Putting Equations (3) and (8) in Equation (7),

(9) $$y^{\left(nj\right)l}=P_{l}g_{\left(n\right)l}^{\left(nj\right)l}\sum_{t=1}^{R_{l}}\frac{{\left[g_{\left(n\right)l}^{\left(nt\right)l}\right]}^{H}}{\left\|g_{\left(n\right)l}^{\left(nt\right)l}\right\|}s^{\left(nj\right)l}+I_{inter-cell}+I_{inter-tier}+z.$$

The intra-cell interference is as follows

(10) $$I_{intra-cell}=P_{l}g_{\left(n\right)l}^{\left(nj\right)l}\sum_{t=1,t\neq j}^{R_{l}}\frac{{\left[g_{\left(n\right)l}^{\left(nt\right)l}\right]}^{H}}{\left\|g_{\left(n\right)l}^{\left(nt\right)l}\right\|}s^{\left(nj\right)l}$$

Hence, the received signal,

(11) $$y^{\left(nj\right)l}=P_{l}\|g_{\left(n\right)l}^{\left(nj\right)l}\|s^{\left(nj\right)l}+I_{intra-cell}+I_{inter-cell}+I_{inter-tier}+z.$$

The first term of the r.h.s. of Equation (11) is the weighted value of the intended signal. The signal power to interference plus noise power ratio (SINR) experienced at the receiver is given by the following:

(12) $$SINR=\frac{{\left(P_{l}\|g_{\left(n\right)l}^{\left(nj\right)l}\|s^{\left(nj\right)l}\right)}^{2}}{I_{total}^{2}+\sigma_{z}^{2}}.$$

Here, $I_{total}=I_{intra-cell}+I_{inter-cell}+I_{inter-tier}$.

### 2.4. Performance Metrics

In this work, two fundamental performance metrics are considered as evaluation parameters of the network performance [27].

#### 2.4.1. Coverage Probability (*CP*)

*CP* is the measure of the reliability of a typical transmission link, and is defined as the probability that a typical mobile user is able to achieve some threshold SINR (*Th*), given by the follwing

(13) CP=Pr[SINR>Th].

For successfully running any given application, the user needs to have an *SINR* value of more than a minimum value. If the *SINR* experienced by any user drops below the desired minimum value, the customer satisfaction would be compromised. Hence, a higher value of *CP* implies a better quality of experience (QoE).

#### 2.4.2. Area Spectral Efficiency (*ASE*)

*ASE* is a measure of spectral reuse efficiency in the network and is defined as the sum of the average data rates per unit bandwidth normalized with the total service area (bits/sec/Hz/Km^2^), given by the following,

(14) $$ASE=\frac{\sum_{k}L_{k}R_{k}}{A}E[{log}_{2}\left(1+SINR^{\left(nj\right)k}\right)].$$

A higher value of *ASE* implies a higher achievable sum rate for the network, which allows a greater number of users to get service from the network.

## 3. Performance Prediction Model

Before deploying a complex HetNet with each tier supporting massive MIMO, network providers are interested in predicting the overall network performance. The analysis provided in the previous section shows that the network performance metrices are functions of various network parameters, such as the number of transmitting antennas at BSs, the number of active users associated per cell, and the transmitted power of each tier. However, the numerical values of the parameters are stochastic variables because of the stochastic network topology and user location. Moreover, for a given topology, the instantaneous performances of the network are also stochastic processes that depend on the stochastic behavior of multipath channel fading coefficients.

In this work, a two-tier network (K = 2) is considered—microcell and picocell. In the given scenario, the numerical values of the network performance metrics (PMs), either CP or ASE, are considered to be stochastically influenced by the number of the antennas present in the BS of both tiers, i.e., $N_{macro}$ and $N_{pico}$; the number of active users served in same time–frequency grid, i.e., $R_{macro}$ and $R_{pico}$, for both tiers; and the difference in the transmitted powers of the tiers in dB, i.e., $P_{TD}=10\;log\left(\frac{P_{macro}}{P_{pico}}\right)$. Thus, the performance metrics are given by the following

(15) $$PM=f\left(N_{macro},R_{macro},N_{pico},R_{pico},P_{TD}\right)+\epsilon .$$

Here, f(.) represents an unknown stochastic function and ϵ represents random noise terms that capture the contributions of the unknown parameters that influence the performance metrics. The objective of the present work is to infer supervised learning models to approximate the unknown stochastic functions for predicting the numerical values of the network performance metrics (PMs). The process is implemented with the following steps, listed below.

### 3.1. Step 1: Data Preparation

In order to accurately predict the performance metrics for an arbitrary network configuration, the supervised learning model requires a quality dataset that is a collection of instances with input attributes and a leveled output. For the given configuration, the input attributes are $N_{macro},R_{macro},N_{pico},R_{pico},P_{TD}$ and the output is (PMs; either CP or ASE). The data set is created by running a realistic simulation for a massive MIMO HetNet system with various combinations of network parameters. For a given set of network parameters (input attributes), the simulation is carried out to evaluate the numerical values of the performance metrics given in Equations (13) and (14). The values of the network parameters and the simulated result of the performance metrics constitute a single instance of the dataset.

**Figure 2:**
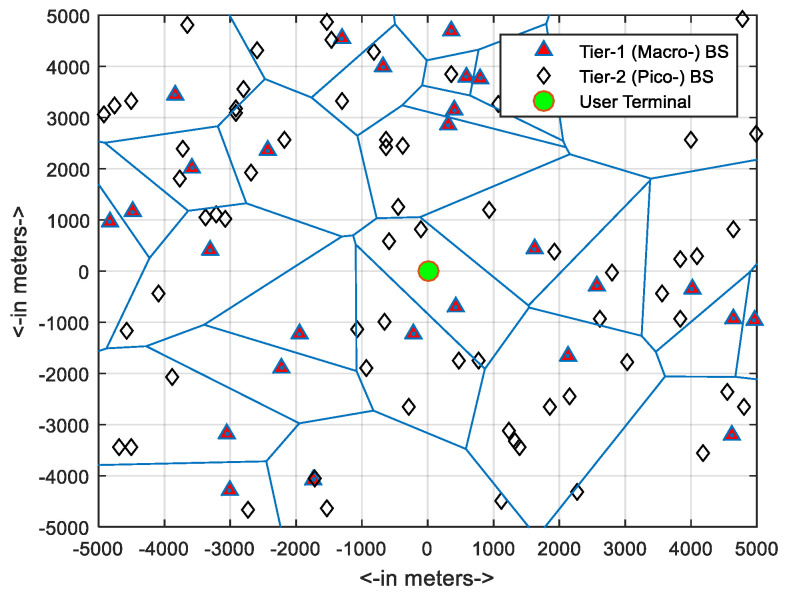
Topology of a simulated two-tier typical network.

In each round of simulation with new input attributes, two-tier cellular networks (K = 2) were simulated, comprising macro-cells and pico-cells, where the BSs of each tier were massive-MIMO enabled. The threshold (Th) of the SINR value in the CP calculation was taken as 10dB. To capture the stochastic nature of the network topology, for each network configuration, the network was simulated 200 times and the results were averaged out. Each time, the cellular network was deployed on a torus of (10 × 10) km^2^. To capture the worst-case scenario, the spatial locations of the BSs stations were modeled using two independent PPPs, with the node densities of two layers related as $\lambda_{pico}=3\lambda_{macro}$. The topology of a typical tow-tier simulated network where the spatial locations of BSs are modeled with two independent PPPs is shown in Figure 2.

For every network topology, the test user was considered to be static, located randomly in the torus. The user was associated to a single BS of any tier that promised the highest average received signal strength as the servicing BS. The signals from all other remaining base stations were considered to be interference. The stochastic channel fading property was captured by simulating each network topology for 1000 coherence time periods with uncorrelated channel coefficients, and the expected values of the outputs were considered. The simulation was carried for 1450 combinations of these network parameters. The ranges of values of all parameters are given in Table 1.

The outputs of the simulation were coverage probability (CP) and area spectral efficiency (ASE) in bits/sec/Hz/km^2^. The output of each combination was recorded as a single instance in the dataset. The prepared dataset was used for model learning and testing.

### 3.2. Step 2: Hypothesis Testing and Model Selection

In the prepared dataset, the initial five columns ($N_{macro},\;N_{pico},R_{macro},\;R_{pico},P_{TD}$), as shown in Table 2, represent the input attributes, and the remaining two are the model target variables (CP and ASE). The dataset was analyzed to evaluate the best fit hypothesis for our problem, stating the statistical relation between the input attributes and the target variables. The null hypothesis for the given problem was set as, “The input attributions are not linearly related with the target”. The existence of a null hypothesis was tested using correlation analysis. Correlation is a measure of the strength and direction of the relationship between variables ranging between −1 to 1. The null hypothesis was established if the correlation coefficient was closely bound to zero, indicating no relation between the variables. Table 3 shows the correlation of the input attributes with the targets.

As the correlation coefficients were not close to zero for any of the inputs, there was sufficient evidence to reject the null hypothesis and to suggest that the PMs (either CP or ASE) showed a linear relationship with the network parameters. Hence, two multivariate linear regression models were inferred to approximate the relations of the network performance metrics (PMs) with the network parameters that will predict the network performance of an arbitrary set of network parameter values outside the training set. Hence, the hypothesis to approximate to an unknown stochastic function is given by the following: [28],

(16) $$\hat{f}=h_{\beta }\left(m\right)=\beta^{T}m.$$

Here, m is the independent input attribute vector given by ${\left[1,N_{macro},\;R_{macro},\;N_{pico},R_{pico},P_{TD}\right]}^{T}$ and $\beta ={\left[\beta_{0},\beta_{1},\ldots ,\beta_{5}\right]}^{T}$ is the tunable regression parameter vector.

### 3.3. Step 3: Training for Best-Fit Model

The model was trained using the dataset with *Q* i.i.d. instances, given by $\chi ={\left\{m^{\left(q\right)},PM^{\left(q\right)}\right\}}_{\left(q\right)=1}^{Q}$, where the *q*-th instance is represented by the superscript $.^{\left(q\right)}$. The loss function for optimization is based on the difference between the simulated output, and the hypothesis evaluation is [20] as follows

(17) $$J\left(\beta |m\right)=\frac{1}{2Q}\sum_{q=1}^{Q}{\left[h_{\beta }\left(m^{\left(q\right)}\right)-PM^{\left(q\right)}\right]}^{2}.$$

The optimal solution corresponds to the values of the regression parameters that minimize the loss function:

(18) $$\hat{\beta }=arg\;\underset{\beta }{min}J\left(\beta |m\right).$$

The optimum solution is obtained using the gradient-descent algorithm, which starts with a random β, and updates it with the given iterative process:

(19) $$\begin{array}{lll} & & \beta_{0}≔\beta_{0}-\frac{\alpha }{Q}\sum_{q=1}^{Q}\left[h_{\beta }\left(m^{\left(q\right)}\right)-PM^{\left(q\right)}\right],\\ & & \beta_{1}≔\beta_{1}-\frac{\alpha }{Q}\sum_{q=1}^{Q}\left[h_{\beta }\left(m^{\left(q\right)}\right)-PM^{\left(q\right)}\right]N_{macro}^{\left(q\right)},\\ Repeat\;until\;convergence & & \{\beta_{2}≔\beta_{2}-\frac{\alpha }{Q}\sum_{q=1}^{Q}\left[h_{\beta }\left(m^{\left(q\right)}\right)-PM^{\left(q\right)}\right]R_{macro}^{\left(q\right)},\\ & & \beta_{3}≔\beta_{3}-\frac{\alpha }{Q}\sum_{q=1}^{Q}\left[h_{\beta }\left(m^{\left(q\right)}\right)-PM^{\left(q\right)}\right]N_{pico}^{\left(q\right)},\\ & & \beta_{4}≔\beta_{4}-\frac{\alpha }{Q}\sum_{q=1}^{Q}\left[h_{\beta }\left(m^{\left(q\right)}\right)-PM^{\left(q\right)}\right]R_{pico}^{\left(q\right)},\\ & & \beta_{5}≔\beta_{5}-\frac{\alpha }{Q}\sum_{q=1}^{Q}\left[h_{\beta }\left(m^{\left(q\right)}\right)-PM^{\left(q\right)}\right]P_{TD}^{\left(q\right)},\} \end{array}$$

Here, α is the step size of the algorithm, taken as 0.01. On convergence, CP and ASE are estimated using Equations (20) and (21), respectively,

(20) $$\hat{CP}={\hat{\beta }}_{CP}^{T}m,$$

(21) $$\hat{ASE}={\hat{\beta }}_{ASE}^{T}m.$$

Here, ${\hat{\beta }}_{CP}$ and ${\hat{\beta }}_{ASE}$ are the regression parameter vectors after convergence for ASE and *CP*, respectively.

### 3.4. Step 4: Model Validation

The acceptability of the hypothesized relationships between variables was evaluated based on the residual errors. The model validation process split the dataset randomly into two sets—the first set consisted of 80% of instances that were used in training the hypothesis, and the second set had the remaining 20% of instances which were used for validating the hypothesis. The generalization ability of the trained regression models is measured using the percentage of error margin and the coefficient of determination, i.e., R^2^ values. To ensure the stability of the trained model, k-fold cross validation was implemented, where the model was evaluated *k* times, with a unique subset for validation. In this work, it was considered to be k = 5. Table 4 provides the five-fold validation evaluation results for both models. The consistency in the evaluation results ensures stability in the models.

### 3.5. Step 5: Final Model Preparation with Complete Dataset

The final model was made to predict the probable output on the new data. The models were finalized by training with the complete available dataset, which was saved for operational use in later cases. In Table 5, the parameter values of the finalized models are tabulated.

**Figure 3:**
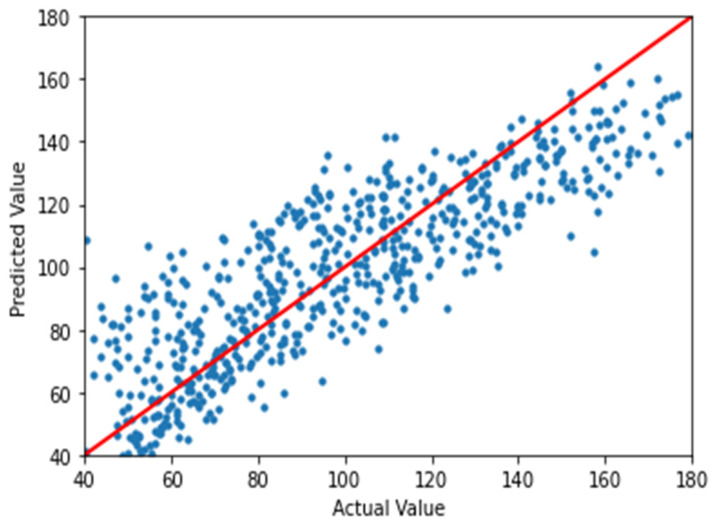
Simulated vs. predicted values of the area spectral density (ASE) in bits/sec/Hz/km^2^ with a trend line.

**Figure 4:**
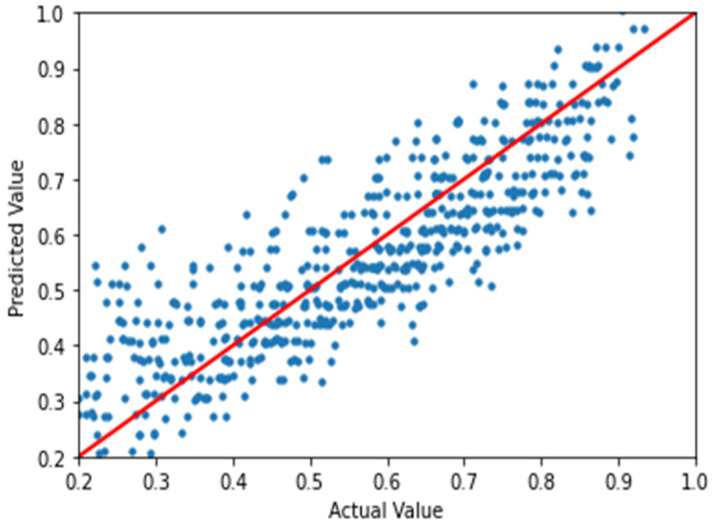
Simulated vs. predicted values of the coverage probability with a trend line.

To visually inspect the performance of the regression process, the actual values vs. predicted values of the finalized model were plotted. Figure 3 and Figure 4 show the actual values vs. values predicted by the finalized model for ASE and CP, respectively, where it could be seen that the model fits linearly with the trend line. The performance of the finalized models has been quantitatively evaluated based on the residue over the predicted output of the dataset, and the results are given in Table 6.

## 4. Conclusions

This paper introduced an ML approach to predict the performance of a MIMO HetNet system considering a multi-cell scenario. The performance metrics considered in this paper are CP and ASE, which are stochastic functions of the network parameters. Two separate multivariate linear regression models have been trained for the network performance metrics, with network parameters as the input attributes. The generalization ability trained models have been evaluated numerically based on the percentage of the error margin and R^2^ score. The error margin for *CP* and *ASE* are found to be 12.06% and 11.32%, respectively, which are within the tolerable range for practical application. The R^2^ scores for *CP* and *ASE* are 0.754 and 0.749, respectively, which are closed to 1, and are found to be satisfactorily high. The visual inspection-based performance evaluation is done using actual vs. predicted output plots. For both models, the scatter points linearly match with the respective trend lines, showing the worth of fitness. During practical deployments of 5G and B5G networks, the application of this model could be very valuable in the precise planning of network and capital expenditures. This model would help the network provider to estimate the quality of service for a given network configuration. The study of other parameters that influence the network performance and their inclusion in the model may be considered as a future direction of research.

## Figures and Tables

**Table 1:** Range of values of all input parameters used in the simulation.

Parameters	Range of Values
$N_{macro}$	50–200
$R_{macro}$	10–40
$N_{pico}$	8–20
$R_{pico}$	4–8
$P_{TD}$	5–20 dB

**Table 2:** Initial five rows of the prepared dataset.

$N_{macro}$	$R_{macro}$	$N_{pico}$	$R_{pico}$	$P_{TD}$	CP	ASE
50	40	20	8	10	0.5786	46.8879
50	40	20	8	20	0.8596	42.4943
100	10	8	4	5	0.4207	60.0825
100	10	8	4	15	0.5017	73.2641
150	10	16	4	5	0.4763	74.8983

**Table 3:** Correlation table.

Attributes	*CP*	*ASE*
$N_{macro}$	0.51	0.59
$R_{macro}$	−0.58	0.48
$N_{pico}$	0.15	0.12
$R_{pico}$	−0.12	−0.11
$P_{TD}$	0.41	0.18

**Table 4:** 5-fold cross validation evaluation results.

Model	Evaluation Parameters	k = 1	k = 2	k = 3	k = 4	k = 5
*CP*	% of error margin	13.18	13.46	13.62	12.08	12.90
	R^2^ score	0.7355	0.749	0.7272	0.7797	0.7624
*ASE*	% of error margin	13.47	12.84	12.35	13.19	13.31
	R^2^ score	0.6782	0.692	0.7274	0.6917	0.7531

**Table 5:** Parameter values of the finalized models.

Coverage Probability	Area Spectral Efficiency
${\hat{\beta }}_{0}^{CP}$ = +0.355020	${\hat{\beta }}_{0}^{ASE}$ = −12.3185
${\hat{\beta }}_{1}^{CP}$ = +0.001934	${\hat{\beta }}_{1}^{ASE}$ = +0.4574
${\hat{\beta }}_{2}^{CP}$ = −0.010107	${\hat{\beta }}_{2}^{ASE}$ = +1.8500
${\hat{\beta }}_{3}^{CP}$ = +0.008203	${\hat{\beta }}_{3}^{ASE}$ = +1.0308
${\hat{\beta }}_{4}^{CP}$ = −0.016797	${\hat{\beta }}_{4}^{ASE}$ = −2.5567
${\hat{\beta }}_{5}^{CP}$ = +0.013265	${\hat{\beta }}_{5}^{ASE}$ = +1.1783

**Table 6:** Evaluation of the finalized models.

Evaluation Parameters	*CP*	*ASE*
% of error margin	12.06	11.32
R^2^ score	0.754	0.749

## Data Availability

No new data were created or analyzed in this study. Data sharing is not applicable to this article.
